# Risk assessment of trace metals in Mefou River sediments, West-Africa

**DOI:** 10.1016/j.heliyon.2021.e08606

**Published:** 2021-12-14

**Authors:** Noa Tang Sylvie Désirée, Ekoa Bessa Armel Zacharie, Messina Thérèse Raïssa, Onana Vincent Laurent, Ndjigui Paul-Désiré

**Affiliations:** a Department of Biological Sciences, Higher Teacher Training School, University of Yaoundé I, Cameroon; b Department of Earth Sciences, Faculty of Science, University of Yaoundé I, Cameroon

**Keywords:** Mefou river, African metropolis, Pollution, Trace metal, Pollution indices

## Abstract

This study focuses on risk assessment of Cr, V, Ni, Co, Pb, Cu, and Zn in the Mefou River sediments located at Yaoundé, West-Africa. Sediment samples were collected from five stations in the downstream of the Mefou River, which drains the urban area of Yaoundé between latitudes 3°30′ and 3°58′ North and longitudes 11°20′ and 11°40′ East. The geochemistry data were analyzed statistically and the pollution indices were calculated in order to identify and estimate the sources of metal contamination in the Mefou River sediments. The results obtained show that the average concentrations of trace metals are almost higher than those of the upper continental crust (UCC) and the metal average in the Simbock Lake cores. However, the concentrations of Ni, Cu, Pb, and Zn in sediments located in most urbanized sites are lower than those of the UCC and the average of Simbock Lake sediments. The pollution indices such as enrichment factor (EF), geo-accumulation index (Igeo), and pollution load index (PLI) showed that trace metals were mainly influenced by human sources, except for Pb, Cu, and Zn, which stemmed from natural sources. The sediments of the Mefou River would therefore be affected with low to moderate pollution levels. The low values of potential ecological risk (RI: 22.36–41.53) suggest a low potential ecological risk effect. The multivariate statistical analysis indicates that Ni, Cu, Pb, Co, and Zn have been derived mainly from natural sources, while V and Cr would partially derive from human activities. The results of this research can be a reference for trace metal pollution along an African urbanized river corridor. This can be considered as an act of prevention of urban watercourses in Cameroon and in other parts of the world, especially in major African urban metropolis.

## 1 Introduction

Environmental pollution is a long-standing problem in modern societies. Among various types of pollution, the considerable contamination of aquatic systems by toxic substances such as trace metals is of great concern because these elements are not biodegradable and their high absorption by agricultural crops can also affect food quality and safety. Trace metals are transferred from land into rivers, lakes and marshes through natural inputs such as erosion and weathering as well as anthropogenic sources, including industrial and agricultural activities, land runoff and wastewater disposal (Ekoa Bessa et al., 2018a; Lone et al., 2018; Mandeng et al., 2019; Shah et al., 2020; Anaya-Gregorio et al., 2018; Ayala-Pérez et al., 2021). Metals released in aquatic systems are often stored in stream sediments through processes such as flocculation, adsorption, and precipitation. As a result, stream sediments are a sink for metals, which can be released through various remobilization processes (Marchand et al., 2006; Barakat et al., 2012). Several studies have demonstrated that metal concentrations in sediments are related to the presence of contaminants in aquatic systems (Suthar et al., 2009; Xiong et al., 2016; Armstrong-Altrin et al., 2019; Ekoa Bessa et al., 2020). Most rivers that flow through densely populated areas are highly vulnerable to trace metals loading from urbanization and industrialization. Anthropogenic impact and natural processes can deteriorate sediment and aquatic environments. Therefore, many scientific works on heavy metals have been carried out over the last decades (Dixit and Tiwari 2008; Jayaprakash et al., 2010; Armstrong-Altrin and Machain-Castillo, 2016; Tehna et al., 2019; Mapel-Hernández et al., 2021). Numerous approaches have been applied to assess the degree of sediment contamination and to understand the natural and anthropogenic inputs to watercourses. Metals assessment indices, such as enrichment factor (EF), geo-accumulation index (Igeo), contamination factor (CF) and pollution load index (PLI) have often been used to assess the degree of sediment contamination (Ekoa Bessa et al., 2018a, 2020; Mandeng et al., 2019).

In Africa and particularly in Cameroon, most industrial and domestic waste water from urban and rural areas are discharged into the environment without prior treatment. Rivers and streams directly receive about 50% of the total pollution caused by human activities (Ekengele et al., 2008). Recent studies on the pollution of aquatic systems by trace metals in Cameroon are mostly focused in mining areas and rarely on rivers in rural or urban areas across the country (Ekengele et al., 2008; Tehna et al., 2015; Mimba et al., 2018; Ekoa Bessa et al., 2018a, b; Mandeng et al., 2019; Tehna et al., 2019). There are several punctual sources of industrial pollution in Cameroon. But at the scale of large hydrosystems, the problems of sediment pollution seem to be mainly due to the lack of sewerage systems. Inadequate water supply is equally one of the main causes of poverty in developing countries, because it affects human health, food security and basic livelihoods. WHO estimates that nearly 80% of the diseases that affect people around the world are directly or indirectly related to water.

This study reports current trace metal distributions and concentrations in the Mefou River sediments and examines the environmental status, sources of pollution and ecological risk in this river located in an urbanized area of southern Cameroon, and by extension in under developing countries around the world.

## 2 Materials and methods

### 2.1 Study area

**Figure 1:**
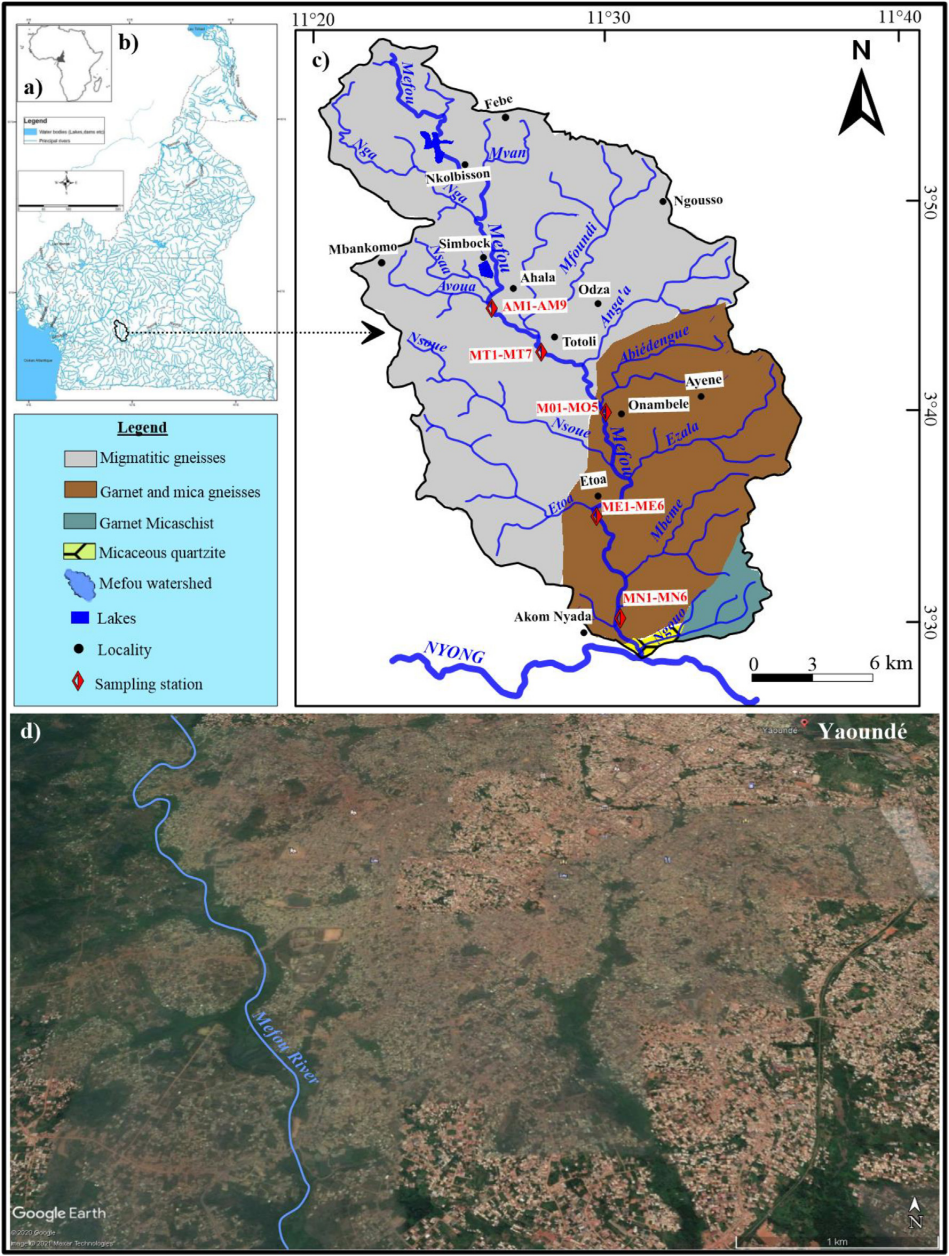
Investigated area and sampling location: a) Location of Cameroon in Africa; b) Location of the study area in Cameroon; c) Mefou watershed; d) Mefou River in Yaoundé and its urban area.

The Mefou watershed is located in Yaoundé, a highly urbanized environment (Figure 1a). This catchment area covers an area of about 840 km^2^ with Mefou as the main river (Figure 1b). The study area which is located in the South Cameroonian Plateau and whose average altitude is 750 m is characterized by a tropical to equatorial transitional climate with four seasons unevenly distributed throughout the year (Ndam et al., 2007). This river is a tributary of order 4 that flows into the Nyong River. The Mefou River takes its source at 800 m of altitude in the Mban-Mimkom massifs. Their main tributaries are the Etoa, Nsaa, and Mfoundi rivers. The watershed is composed by metamorphic series typical of the Yaoundé geological group. This group consists of the metamorphic series of Yokadouma, Mbalmayo, Bengbis, Ayos, and Yaoundé (Nédélec et al., 1986). The Yaoundé series consists of two major lithological units, notably a meta-sedimentary unit consisting of garnet and kyanite gneisses, garnet and plagioclase gneisses and a meta-igneous unit consisting of garnet pyribolites, pyroxenites, biotitites and pyriclastites (Nédélec et al., 1986). The composition of gneisses and silico-calcic rocks is similar to that of a sedimentary sequence that contains pelites, grawackes, dolomites, evaporites and iron-rich sediments (Nzenti, 1998). The soils are mostly red or yellow ferralsols developed on metamorphic rocks (Ndjigui et al., 2013), and gleysols occur in the valleys (Ekoa Bessa et al., 2018b).

### 2.2 Sampling and analysis of sediments

#### 2.2.1 Sampling

Five cores were collected along the Mefou River at Totoli, Onambele, Akomnyada, Etoa, and Ahala. The sampling was manually conducted using polyvinyl chloride pipe in a raft as described by many authors (e.g., Ekoa Bessa et al., 2018a, b; N'nanga et al., 2019). For all cores, sampling was done at each 10 cm interval. Thus, seven samples were taken from the core sediments at Totoli (MT1-MT7); five for Onambele (MO1-MO5); six for Akomnyada (NM1-NM6); six for Etoa (ME1-ME6), and nine for Ahala (AM1-AM9).

#### 2.2.2 Trace metals analysis

The geochemical analyses were done at the Ontario Geological Survey's Geoscience Laboratories at Sudbury (Canada). The concentrations of trace metal elements, specifically trace metals such as Cr, V, Ni, Co, Pb, Cu, and Zn were determined by mass spectrometry after tri-acid digestion in closed beakers using the PerkinElmer Elan 9000 ICP-MS. This method requires the modification of three sets of elements commonly used in samples to incorporate or remove major elements from the list. The International Reference Material (INTL) used is GSP-2 and the standard in-house material (IHST) is MRB-29. Al_2_O_3_ concentrations were determined by X-ray fluorescence as described by Mandeng et al. (2019). Al_2_O_3_ concentrations (in wt.%) have been converted into Al concentrations in mg/kg. The International Reference Material (INTL) used is SY-4 and the in-house standard (IHST) is QS-1. The relative error varies between 0.007 and 2%. In addition, the analyses are carried out in two repetitions and blank to ensure the repeatability and reproducibility of the data and the validation of the method and the results.

#### 2.2.3 Organic matter analysis

The thirty-three samples taken from the cores were air dried during 7 days before being crushed. The total organic carbon was determined in fifteen samples (three samples per station, in the base, middle and the top of the cores) using a Primacs SLC analyser at the laboratories of the International Institute for Tropical Agriculture (IITA; Yaoundé, Cameroon). The amount of organic matter (OM) was obtained by the formula OM (%) = TOC (%) x 1.72.

### 2.3 Pollution indices and ecological risk assessment approaches

Several indices are used to determine the degree of sediment contamination. In this study, five indices were used to assess the degree of trace metal contamination in sediments and the ecological risks they are likely to create in the Yaoundé urban area. In order to compare the obtained values in these sediments, the mean values of the UCC (Taylor and McLennan, 1995) and average data from Simbock Lake in Yaoundé (Ekoa Bessa et al., 2018b) were used as references for all the indices in this study (Table 1).

**Table 1:** Classes of EF,I-geo, RI, and CF in relation to enrichment, pollution, potential ecological risk, ecological risk and contamination levels, respectively.

EF classes	Enrichment level	I-geo value; classes	Pollution level
EF < 1	No enrichment	I-geo ≤ 0; 0	Unpolluted
EF = 1–3	Minor enrichment	I-geo = 0–1; 1	Unpolluted to moderately polluted
EF = 3–5	Moderate enrichment	I-geo = 1–2; 2	Moderately polluted
EF = 5–10	Moderately severe enrichment	I-geo = 2–3; 3	Moderately to strongly polluted
EF = 25–50	Very severe enrichment	I-geo = 3–4; 4	Strongly polluted
EF > 50	Extremely severe enrichment	I-geo = 4–5; 5	Strongly to very strongly polluted
RI classes	Risk level	CF classes	Contamination level
RI < 150	Low ecological risk	CF < 1	Low contamination
RI = 150–300	Moderate ecological risk	CF = 1-3	Moderate contamination
RI = 300–600	Significant ecological risk	CF = 3-6	Considerable contamination
RI > 600	High ecological risk	CF > 6	High contamination

#### 2.3.1 Enrichment factor (EF)

In order to collect information on the sources and spatial distribution of trace metals, the EF values of selected metals in river sediments were calculated using the equation from Zhang and Shan (2008):

(1) EF=(M/Al)sample/(M/Al)background

In Eq. (1), M_sample_ and M_background_ are the contents in metal investigated in the sediment sample and the uncontaminated background respectively; Al_sample_ and Al_background_ are the contents of Al in the sediment sample and the uncontaminated background respectively. The reference values are data from Simbock Lake in Yaoundé (Ekoa Bessa et al., 2018b).

#### 2.3.2 Geo-accumulation index (Igeo)

The geo-accumulation index (Igeo), which was first described by Müller (1969), is widely used to estimate the level of trace metal pollution of sediments in aquatic systems. This method provides a direct measure of the degree of enrichment and accumulation of trace metals in sediments. This index can be calculated as follows:

(2) $$\text{Igeo}=\text{Log}2\left({\text{C}}_{\text{sample}}/{1.5\text{B}}_{\text{sample}}\right)$$

In Eq. (2), C_sample_ is set for the measured metal concentration in the sediment and B_sample_ represents the reference value or geochemical background of a particular trace metal. Al was used as reference in this study due to its high abundance in the crust, fixation in fine sediments, low bioavailability and high stability. The mean values for the reference data from Simbock Lake sediments in Yaoundé (Ekoa Bessa et al., 2018b) were equally used as reference values.

#### 2.3.3 Contamination factor (CF) and pollution load index (PLI)

To assess sediment quality, an integrated approach to the pollution load index of the selected eight metals (Al, Cr, V, Ni, Co, Pb, Cu, and Zn) is calculated according to Tomlinson et al. (1980). The PLI is defined as the nth root of the sum of the contamination factors (CFs) of the studied metals. PLI is calculated as follows:

(3) $$PLI=\sqrt[{n}]{\left(CF1\times CF2\times CF3\times \ldots \ldots \times CFn\right)}$$

where CF is the ratio between the content of each metal and the reference values in the sediment.

(4) $$CF=\left(\frac{C_{metal}}{C_{background}}\right)$$

In Eq. (4), C_metal_ is the concentration of the metal in the sediment and C_background_ is the reference value. CF was classified into four groups (see Table 1) in order to assess the level of contamination (Mmolawa et al., 2011).

#### 2.3.4 Ecological risk assessment

The Potential Ecological Risk index (RI) was proposed by Hakanson (1980) and is used to assess the potential ecological risk that trace metals in sediments may cause (Maanan et al., 2015; Ekoa Bessa et al., 2018b). This method considers four factors namely: concentration, type of pollutant, level of toxicity, and sensitivity of the water body to metal contamination in sediment. The RI index is calculated as follows:

(5) $$RI=\sum_{1}^{n}Er$$

(6) Er=Tr×CF

where Er is the potential ecological risk coefficient, n the class of metal selected, and Tr, the toxic response factor suggested by Hakanson (1980) for five metals: Cr = 2, Ni = 5, Pb = 5, Cu = 5, and Zn = 1. Er (6) and RI (5) express the potential ecological risk factor for each metal and for several metals, respectively. The expressions and values used in the interpretation of the potential ecological risk factor (Hakanson 1980) are shown in Table 1.

### 2.4 Statistical analysis

Pearson correlation analysis was conducted to determine the relationships between trace metals and sampling stations. A factor analysis was performed by evaluating the main components and calculating eigenvectors to determine common pollution sources. Rotation of the main components was performed using the Varimax method. The statistical analysis was performed using XLSTAT version 16 software and the samples were subjected to a one-way ANOVA with replication equal to the probability level of 0.05 (Duncan's test) using the COSTAT 6.3 program (Ekoa Bessa et al., 2020).

## 3 Results

### 3.1 Spatial distribution of metal concentrations

Mean concentrations of selected trace metals (Al, Cr, V, Ni, Co, Pb, Cu, and Zn) and organic matter content are shown in Table 2. Sediments from Mefou River are slightly humid and the grain size varies from sand to clay with a greater proportion of sand. Organic matter proportions in the materials vary between 1.39 and 5.85% (Table 1). These results show that the mean concentrations are generally higher than the UCC average values (Taylor and McLennan, 1995) and local average data (Ekoa Bessa et al., 2018b) that were used as reference values, although some exceptions were observed at some stations. For example, Ni, Pb, and Zn concentrations are below the reference values at the Ahala station, while copper is below the reference value at the Etoa station. At the Onambele station, Zn has values that are below the reference values (Table 2). These values are similar to those found previously in Simbock Lake in Yaoundé, which is fed by the same watercourse (Ekoa Bessa et al., 2018b). The values obtained in this study are also similar to those obtained by Barakat et al. (2012) in the surface sediments of the Day River (Morocco). These values are opposed to those in sediments from two watersheds in the Abiete-Toko gold district, located in southern Cameroon, which is poorly urbanized although it uses toxic substances for artisanal gold mining (Mandeng et al., 2019).

**Table 2:** Mean heavy metal concentrations in mg/kg with their minimal, maximum and standard deviation in sediments from Mefou River.

Elements		Al	Cr	V	Ni	Co	Pb	Cu	Zn	OM
Toutouli (n = 7)	Min.	46440.16	62.00	80.80	12.50	10.39	8.87	6.60	38.00	3.78
	Max.	156246.00	127.00	176.40	67.90	48.37	40.67	49.00	163.40	4.25
	Mean	90308.08	92.67	120.91	34.68	26.30	20.39	23.11	85.82	4.02
	SD	37553.21	24.46	33.88	18.67	13.80	10.98	14.50	42.12	0.33
Onambele (n = 5)	Min.	41404.48	62.00	64.90	10.40	8.33	10.34	6.90	30.20	1.39
	Max.	133445.50	136.00	162.50	48.00	18.45	39.06	42.20	126.30	3.81
	Mean	85856.35	103.43	114.27	28.53	13.74	23.21	22.77	70.56	2.60
	SD	34471.89	27.98	41.21	13.34	4.12	10.82	14.34	35.75	1.71
Akomnyada (n = 6)	Min.	61337.38	73.00	77.40	16.80	3.04	15.52	8.50	34.50	3.12
	Max.	137082.40	164.00	200.70	51.90	58.52	33.20	23.20	94.40	5.10
	Mean	91752.54	104.25	120.31	29.83	17.46	21.66	13.89	53.31	4.11
	SD	25858.31	32.45	44.13	12.15	22.29	6.28	5.09	22.20	1.40
Etoa (n = 6)	Min.	115890.60	147.00	160.40	43.70	41.07	25.43	15.80	73.80	4.02
	Max.	160302.50	203.00	192.70	66.50	95.61	62.87	41.10	174.80	5.85
	Mean	140177.30	178.00	176.34	55.48	63.96	42.03	26.38	121.18	4.94
	SD	16877.75	22.55	15.62	9.38	24.77	16.65	10.86	39.87	1.39
Ahala (n = 9)	Min.	56511.52	85.00	79.00	4.00	4.04	3.52	1.40	19.20	3.89
	Max.	293118.50	640.00	258.00	42.50	28.80	22.05	32.80	95.50	5.26
	Mean	106099.00	259.55	170.82	18.27	14.09	11.71	14.54	46.24	4.58
	SD	69424.20	176.34	59.73	12.64	8.11	7.68	11.91	24.16	0.97
UCC∗	-	80400.00	83.00	107.00	44.00	17.00	17.00	25.00	71.00	-
Reference data∗∗		68491.42	145.00	133.00	36.00	25.08	17.68	38.00	83.00	-

SD: Standard deviation.

∗ Average concentrations of the upper continental crust (Taylor and McLennan, 1995).

∗∗ local reference data from Simbock Lake cores (Ekoa Bessa et al., 2018a, b).

**Figure 2:**
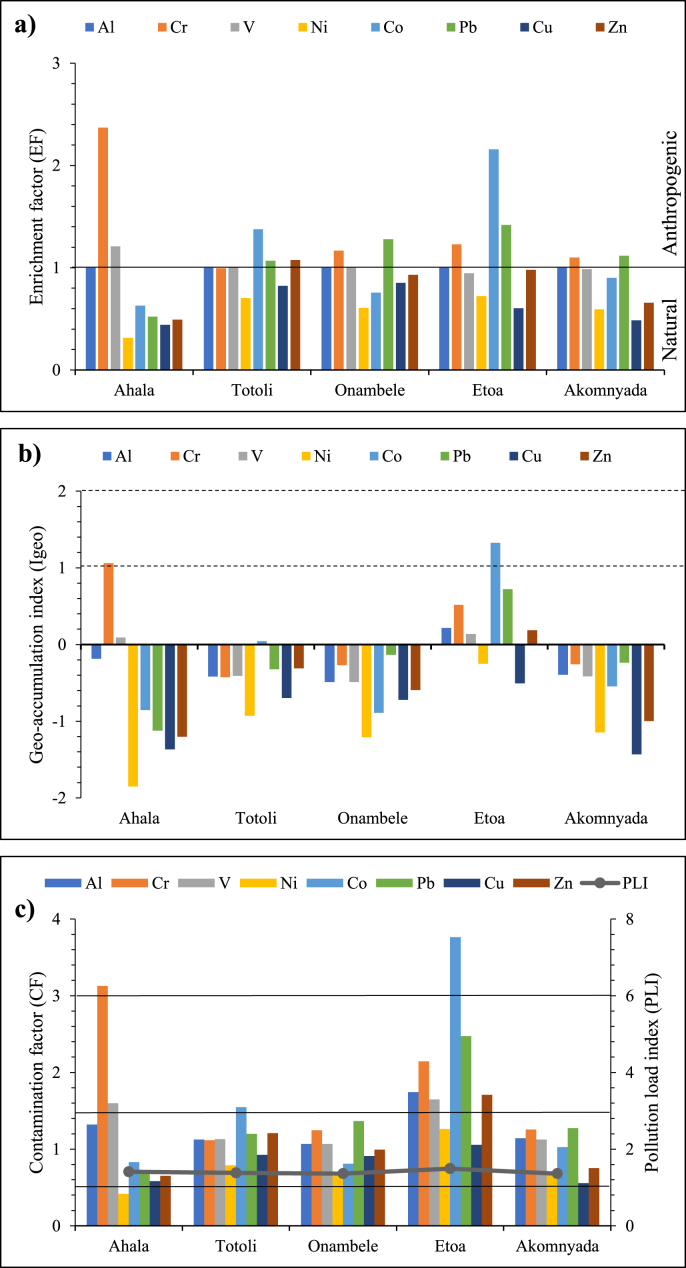
Pollution indices: a) Enrichment factor of heavy metals in the sediments from Mefou River; b) geo-accumulation indexof selected metals in the sediments from Mefou River; c) contamination factor (CF) and pollution load index (PLI) of heavy metals in the Mefou River sediments.

Figure 2c shows the result of the hierarchical cluster analysis of sampling stations based on data of metal concentrations in sediments. Two main groups of different samples were observed. The first contained several stations along the Mefou River indicating heavily polluted samples and stations; the second group was composed of the remaining stations and represented the less polluted stations and samples. The results of the statistical analysis indicated that the concentrations of Al, Cr, and V were significant with a probability level of 0.05, for all selected stations grouped by hierarchical cluster analysis. Contaminants from point sources could be responsible for the highly polluted stations, whereas those from non-point sources could be responsible for the less polluted stations.

### 3.2 Enrichment factor

To accurately assess the anthropogenic influences on the trace metals in the sediments from the Mefou River, the EF for each metal was calculated and listed in Table 3 and Figure 2a. The sequence of the mean EF values in the studied sediments decreases as follows: Cr (3.25) > Co (1.98) > V (1.84) > Ni (1.29) >Pb (0.92) > Zn (0.83) > Cu (0.64). The spatial distribution pattern of the EF values of the selected metals is similar to that of their contents. Table 1 shows the range values and the interpretations of results from different calculations. In the Mefou River, the mean EF values of Pb, Cu and Zn are all below 1.5, indicating that these metals would come from natural sources (Zhang and Liu, 2002), such as weathering and erosion of surrounding rocks.

**Table 3:** Pollution indices (EF, Igeo, CF and PLI) and ecological risk assessment (Er and RI) Enrichment Factor (EF).

Sites	Element								
	Al	Cr	V	Ni	Co	Pb	Cu	Zn	
Toutouli	1	0.99	1.01	0.70	1.38	1.07	0.82	1.08	
Onambele	1	1.17	1.00	0.61	0.76	1.28	0.85	0.93	
Akomnyada	1	1.10	0.99	0.59	0.90	1.12	0.49	0.66	
Etoa	1	1.23	0.95	0.72	2.16	1.42	0.61	0.98	
Ahala	1	2.37	1.21	0.31	0.63	0.52	0.44	0.49	

Geo-accumulation index (Igeo)									

Element	Al	Cr	V	Ni	Co	Pb	Cu	Zn	

Toutouli	-0.42	-0.43	-0.41	-0.93	0.04	-0.32	-0.70	-0.31	
Onambele	-0.49	-0.27	-0.49	-1.21	-0.89	-0.14	-0.72	-0.59	
Akomnyada	-0.39	-0.26	-0.42	-1.15	-0.55	-0.24	-1.43	-1.00	
Etoa	0.22	0.52	0.14	-0.25	1.33	0.72	-0.51	0.19	
Ahala	-0.18	1.06	0.09	-1.85	-0.86	-1.12	-1.37	-1.20	

Contamination factor (CF) and pollution load index (PLI)									
Element	Al	Cr	V	Ni	Co	Pb	Cu	Zn	PLI
Toutouli	1.12	1.12	1.13	0.79	1.55	1.20	0.92	1.21	1.38
Onambele	1.07	1.25	1.07	0.65	0.81	1.37	0.91	0.99	1.36
Akomnyada	1.14	1.26	1.12	0.68	1.03	1.27	0.56	0.75	1.36
Etoa	1.74	2.14	1.65	1.26	3.76	2.47	1.06	1.71	1.49
Ahala	1.32	3.13	1.60	0.42	0.83	0.69	0.58	0.65	1.41
Ecological risk factor (Er) and ecological risk index (RI)									

Element	Cr	Ni	Pb	Cu	Zn	RI			

Toutouli	5.30	8.67	5.10	4.62	1.21	24.89			
Onambele	5.91	7.13	5.80	4.55	0.99	24.39			
Akomnyada	5.96	7.46	5.42	2.78	0.75	22.36			
Etoa	10.17	13.87	10.51	5.28	1.71	41.53			
Ahala	14.83	4.57	2.93	2.91	0.65	25.89			

### 3.3 Geo-accumulation index

The results of the geo-accumulation index calculation (Table 3; Figure 2b) reveal that the sediments collected from the Mefou River are unpolluted to moderately polluted. Their average values decrease in the following order: Cr (1.31) > V (0.62) > Co (0.58) > Ni (0.06) > Al (−0.25) >Pb (−0.45) > Zn (−0.58) > Cu (−0.95). These results show that all the stations are polluted in Cr and V on the one hand and all these stations are not polluted by Al, Pb, Cu, and Zn except for the Etoa station. In Etoa station, all selected trace metals are polluted except for Cu. The majority of elements in this study are of class 1 according to the classification of Müller (1969) except for Cr which is of class 2 at the Etoa station indicating a moderate pollution in this element. Similarly, Cr at the Ahala station and Co at Etoa station are of class 3 indicating a moderate to heavy pollution (Figure 2b).

### 3.4 Contamination factor and pollution load index

According to Hakanson (1980) classification, the CF of the majority of metals in the Mefou River sediments, except for Cu and Zn (1 < CF < 3) showed a low contamination factor at many stations except for Totoli and Etoa (Table 3; Figure 2c). Chromium has a significant degree of contamination at the Etoa station (3 < CF < 6) and a high degree at the Ahala station (CF > 6), as it is the case with Co, which has a high degree of contamination in the sediments of Etoa (CF > 6). PLI represents the number of times the metal content in the sediment exceeds the concentration of the reference values and gives a summative indication of the overall level of trace metal toxicity in a given sample. For PLI >1, the station is polluted, whereas PLI< 1 indicates an absence of pollution. The PLI values obtained in the sediments of the Mefou River are summarized in Table 3. These values range from 1.36 to 1.49, with the Etoa and Ahala stations being the most polluted (Figure 2c). This indicates that the concentrations of Cr, Co, V, Ni, Pb, Zn, and Cu at most stations exceed the reference values.

### 3.5 Potential ecological risk

Potential ecological risk represents the sensitivity of the biological community to a given substance and illustrates the risk caused by contamination (Hakanson, 1980). The potential ecological harm of a single trace metal and the potential ecological risk index (Er) of several trace metals (Eqs. (5) and (6); Table 3) were calculated and the results indicate that the majority of the studied metals contributed to the ecological risk that may be induced in the sediments of the Mefou River. Er values for Cr, Ni, Pb, Cu, and Zn in Mefou River sediments are below 40, thus suggesting a low ecological risk (Table 3). Based on the potential ecological risk (RI) values (Table 3), all samples present a low ecological risk. It is therefore important to continue to carefully monitor trace metal contaminants, particularly Cr, V, and Co in fluvial sediments in the Mefou watershed.

### 3.6 Principal component and cluster analyses

**Figure 3:**
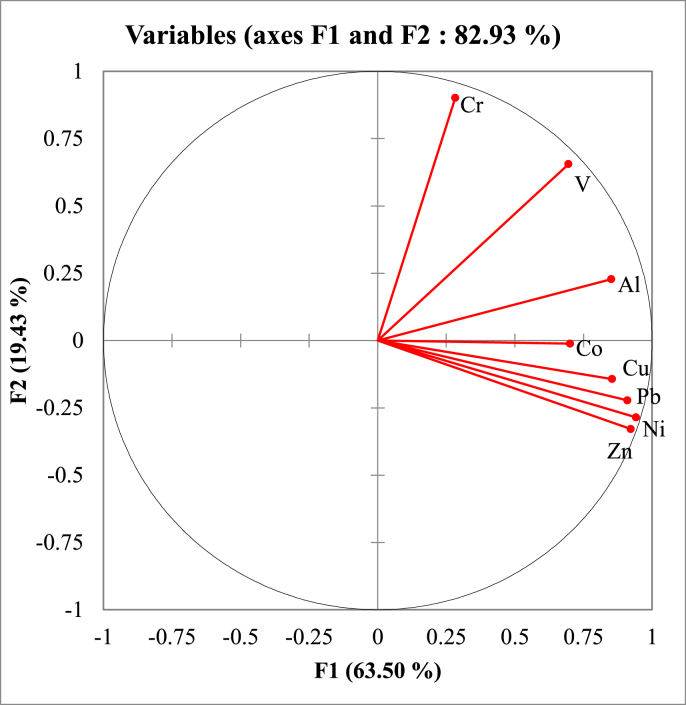
Principal component analysis of studied metals from sampling stations along the Mefou River.

**Figure 4:**
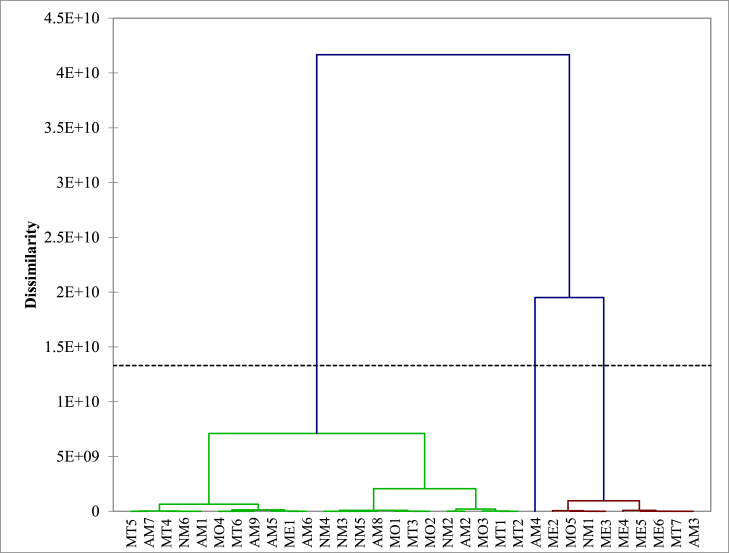
Dendrogram showing clustering of the sampling locations in the Mefou River.

Principal Component Analysis (PCA) showed that the eight trace metals in the sediments of the Mefou River can be described using two principal components that explain 63.50% (F1) and 19.43% (F2) for a cumulative contribution rate of 82.93% (Figure 3). For the first main component (F1), all studied metals were found to have significant loadings, corresponding to their high levels in sediments in relation to baseline values and significant correlations. The second component (F2) shows a negative correlation for all metals except for V, Cr and Al (Figure 3). Therefore, these metals were probably not influenced by anthropogenic factors, but rather by natural sources such as weathering and erosion of surrounding rocks which can be group into 3 main sources (Figure 4). In addition, the results showed that three metals (Al, Cr, and V) were mainly distributed in sediments near heavily urbanized areas.

## 4 Discussion

### 4.1 Correlation analysis

The relationships between metals were analyzed using the Pearson correlation matrix with *P* significance <0.05 (Table 4). To identify the common characteristics (behavior, origin, etc.) of metals in Mefou River sediments, metal correlation analyses were calculated. In these sediments, the correlations between the metals Al, Cr, Co, V, Ni, Pb, Zn, and Cu are strongly positive with a probability level p < 0.05 (Table 4), except for the correlation of Cr and Zn. This suggests that the source would be similar for most of the metals. Aluminum is positively correlated with all metals except Cr. This could also justify its use as a reference element. The strong positive correlations between some metals (Zn vs. Ni, Zn vs. Pb, Ni vs. Pb, and Ni vs. Co) suggest a similar geochemical behavior or same origin. Factors such as source rock types, weathering and erosion processes, surface absorption phenomena and the characteristics of the deposition environment influence the distribution of metals in the sediments of the Mefou River. Conversely, the positive correlations between V and Cr could be explained by a common origin mainly related to local sources of pollution points such as solid and liquid discharges. Vanadium and Cr seem also well correlated to the other metals and could possibility be linked to the lithology and grain size.

**Table 4:** Correlation matrix of heavy metals in the Mefou river sediments.

Variables	Al	Cr	V	Ni	Co	Pb	Cu	Zn
Al	**1**							
Cr	0.36	**1**						
V	**0.74**	**0.72**	**1**					
Ni	**0.72**	0.02	**0.47**	**1**				
Co	**0.54**	0.13	**0.50**	**0.71**	**1**			
Pb	**0.71**	0.12	**0.45**	**0.91**	**0.53**	**1**		
Cu	**0.61**	0.18	**0.49**	**0.82**	0.38	**0.81**	**1**	
Zn	**0.70**	-0.02	**0.42**	**0.95**	**0.59**	**0.91**	**0.87**	**1**

Values in bold are significantly different from 0 at significance level alpha = 0.05.

It is likely that urban pollution contributed to the accumulation of Cr and V in the sediments of the Mefou River (Saiful Islam et al., 2015; Ekoa Bessa et al., 2018b).

### 4.2 Assessment of sediment contamination

Numerous indices are available to infer the environmental contamination with heavy metals in sediments, which are based on metal content, bioavailability and toxicity (Yang et al., 2009). The interpretations of this study are similar to the sediments of a river in a sparsely urbanized area of southern Bangladesh, collected during the dry season (Saiful Islam et al., 2015). It is suggesting that the more an area is urbanized, the more it is exposed to metals contamination or pollution. However, Cr which is the most abundant elements after Al, has values above 1.5 at Ahala and Co at Etoa on the Mefou River. Based on the classification of enrichment levels (Table 1) established by several authors (Hakanson, 1980; Soltani et al., 2015), the results indicate that the sediments of the Mefou River would have a minor enrichment of the selected trace metals, while Cr would have a moderate enrichment at the Ahala station and Co at the Etoa station. This is consistent with a previous study conducted in the Simbock Lake (Ekoa Bessa et al., 2018b) fed by the Mefou River further upstream of the study stations. These results are also similar to those obtained by some related studies in Morocco (Barakat et al., 2012) and India (Chabukdhara and Nema, 2012).

The EF values obtained during this study may be useful indicators of the role of anthropogenic processes in their distribution. Pollution of Cr, V and some other metals in some stations would result from the incineration of car wheels, household waste, pesticide use and abandoned metal alloy that end up in the Mefou catchment area and are transported to the main collector which is the Mefou River (Ekoa Bessa et al., 2018b; Mbale et al., 2019). The sediments of the Mefou River have also been globally polluted by these metals as a result of anthropogenic activities in the Mefou catchment. Otherwise, Cr, Co and other metals with higher to low levels of contamination at most stations are clearly associated to local sources of pollution such as solid and liquid discharges in the city of Yaoundé and its surrounding areas (Ekoa Bessa et al., 2018b).

### 4.3 Assessment of ecological risk

The Er and RI of the heavy metals in the investigated cores showed low potential ecological risk (Er < 40). It is clear that all the cores showed low ecological risk index. This indicates low polluted according to Hakanson (1980). On the other hand, the average concentrations of metals in this study are sometime higher than the reference values. These abnormal values can be attributed to the result of natural weathering and leaching of rocks and the pollutant load provided by the various discharges from agricultural and artisanal mining activities along the watershed.

Aluminum is one of the most abundant elements in the earth's crust. This element is naturally present in sediments, water and nutritious food. The presence of Al in the body is of concern because it is suspected to increase the risk of certain cancers (Fashola et al., 2016). In this study, Al concentrations were higher than that of other metals at all sampling stations, with mean concentrations ranging from 85856.35 to 140177.3 mg/kg. This indicates that this metal is naturally present in large quantities in sediments. However, the highest concentrations are located at the exit of Yaoundé.

Chromium is found in metamorphic rocks where it easily substitutes iron, which has an ionic radius close to that of Cr (III). It is also found in sedimentary and meta-sedimentary rocks. Chromium (III) occurs naturally in the environment and it is an essential trace element, particularly for the metabolism of sugar, proteins and fat in humans (Abdullah et al., 2015). Chromium derivatives are highly toxic in high doses, its salts are a rapid cause of ulcers. It is one of the toxic metals present in certain industrial waste, incinerator waste, or in certain sediments. Its concentration varies between 92.67 and 259.55 mg/kg. Elevated Cr values were detected at the Ahala and Etoa stations as it was the case with Al, suggesting that the sources of Cr appear to be much more natural than anthropogenic, probably due to the regional geology which is composed of metamorphic and meta-sedimentary rocks and the incineration of car wheels, plastic waste and plant remains.

Vanadium is present in various ores, couple with other metals, in coal and petroleum. In addition to human activities, other sources of waste are product of industrial manufacture in metallurgy (metal alloys, including steel), petrochemicals, bitumen, asphalt, tars, soot and ashes from thermal power stations. The essential trace element character of V has not yet been formally demonstrated. With regard to its toxic effects, V is essentially a lung and eye irritant. It can also cause digestive disorders. Repeated exposure to V derivatives may cause rhinitis, pharyngitis, laryngitis, chronic bronchitis, skin irritation and dark green discoloration of the tongue and skin (Malik et al., 2004). In this study, V values range from 114.27 to 176.34 mg/kg. The highest values are like the previous developed metals located in the Ahala and Etoa stations at the exit of Yaoundé. These high values would be related to anthropogenic activities carried out in the city of Yaoundé and which would decrease as one moves away from the highly urbanized areas, but also naturally due to the action of erosion of surrounding rocks. This could be explained by the fact that during civil engineering works such as road construction, the remains of metal alloy are abandoned and dumped into the city's waterways, and these in turn flow into the main sewer, which is the Mefou River.

Nickel is a very common metal in the environment. It has both natural (weathering of source rocks) and anthropogenic (electroplating, non-ferrous metals, paints and porcelain enamellings) sources. The effects of Ni in the human body are variable and include dermatitis, cardiovascular disease, chest pain, dizziness, kidney disease, dry cough and shortness of breath, nausea, headaches, and lung and nasal cancer (Malik et al., 2004; Fashola et al., 2016). The nickel concentrations in this study are 18.27–55.48 mg/kg, with the highest value the Etoa station. The highest concentrations of Ni at the Etoa station could be attributed to paints and porcelain enamellings, although carried out by craftsmen in the vicinity of this locality. The relatively high values in the other stations could be attributed to the weathering of the surrounding rocks (Ekoa Bessa et al., 2018a, b; Mandeng et al., 2019).

Cobalt is used in metallurgy for certain parts of the reactor or gas turbines. This element is mainly found in soils and sediments. At very low proportion, it is a trace element useful to a few rare plants that know how to fix nitrogen, and seem indispensable to a greater number of fixing bacteria associated with the root sphere of many plants. Average concentrations of Co in the continental crust are about 10 mg/kg (Taylor and McLennan, 1995). This element is generally associated with Ni and behaves similarly. Concentrations of Co found in sediments of the Mefou River range from 13.74 to 63.96 mg/kg (Table 2). This high concentration could be related to the presence of iron oxyhydroxides in the Mefou River sediments, as Co is considered to have low-mobility and strong affinity with iron.

Lead is present in the Earth's crust and in all compartments of the biosphere. Lead is mainly emitted by industrial activities: metallurgy, production of materials and use of non-metallic minerals (Abdullah et al., 2015). Lead can also be emitted from certain paints and thus pollute the air, water and sediments, and consequently plants, animals and food. It is the most recovered and recycled non-ferrous metal. The main sources of Pb are believed to come from the use of organo-metallic compounds such as antiknock agents in combustion engines. For example, it is estimated that 95% of lead air pollution comes from gasoline, mining, metallurgy, battery manufacturing, recycling, leaded paint, electronic scrap, Pb contaminated food and water, and some traditional medicines (WHO 2011). Lead toxicity is high in crops such as tomatoes and rice, leading to physiological and biochemical disorders that reduce photosynthesis and transpiration, resulting in stunted growth (Bazzaz et al., 1974). Lead concentrations range from 11.71 to 42.03 mg/kg with the highest values found at the Etoa station. The high contents of Pb observed at all stations indicate that its source could be urban discharges such as those from garages, tomato cultivation located mainly in Etoa where these activities are carried out on the banks of the Mefou River, and the use of certain traditional medicines by the populations in the cities.

Copper is an essential trace element that is required in small quantities (5–20 mg/kg) for human beings. As a result of its malleability, ductility, and electrical conductivity proprieties, it is used in alloys, fabrication of tools, coins, jewellery, food and beverage containers, automotive brake pads, electrical wiring, and electroplating (Abdullah et al., 2015). In fact, Cu is one of the most toxic metals for aquatic organisms. When a person is exposed to Cu above the essential levels required for good health, the liver and kidneys produce metallothionein (Fashola et al., 2016). Concentrations of Cu are relatively low in this study, ranging from 13.89 to 26.38 mg/kg. The highest Cu content was detected in samples from the Etoa station where the content is higher than those of the continental crust suggesting an anthropogenic contribution due to the proximity to garages and metal workshops.

Zinc is a moderately reactive metal that combines with oxygen and other non-metals and reacts with dilute acids to release hydrogen (Malik et al., 2004). Natural sources of Zn in the environment include rock weathering (56%), volcanism (22%) and vegetation (Horowitz, 1985). The main use of Zn is in the galvanization of steel, which consumes 47% of Zn mined worldwide and is used in automotive, construction, household appliances, industrial equipment, and other applications including roofing plates, parts and the cultivation of corn. The human physiological disorders caused by this type of intoxication in a primary case will include nausea and disorders of the gastrointestinal system, followed by complications in the respiratory system and skin disorders; Zn is suspected to be carcinogenic to humans (Fashola et al., 2016). Concentrations of Zn in the sediments of the Mefou River range from 46.24 to 121.18 mg/kg. Higher concentrations of Zn are recorded at the Etoa station. The high Zn values are believed to be related to corn cultivation and the presence of garages in the vicinity of the Mefou River.

## 5 Conclusions

This research reveals that urban development negatively influences the accumulation of trace metals in the sediments of the Mefou River. The most important findings are as follows:

1. Multivariate statistical analysis showed that all the studied metals have significant loads, corresponding to their high contents in the sediments as compared to reference values, and all the studied trace metals have been influenced by natural sources such as weathering and erosion of surrounding rocks.
2. The mean values of Al, Cr, V, Ni, Co, Pb, Cu, and Zn are higher than that of the UCC and the reference data from Simbock Lake in urbanized area of Yaoundé, and the highest values for Al, Cr, V, and Co are found near the most urbanized areas. The high Al, Cr, V, and Co concentrations are due to anthropogenic activities along the Mefou River while Pb, Cu, and Zn have a natural origin.
3. The ecological risk assessment showed that the majority of the studied metals contributed to the ecological risks induced by the sediments of the Mefou River. The low values of the ecological risk indices suggest a low potential ecological risk.
4. The sediments of the Mefou River are slightly polluted by trace metals. To solve this situation, the need of waste water treatment plants by local authorities is necessary.

## Declarations

### Author contribution statement

Noa Tang Sylvie Désirée: Conceived and designed the experiments; Performed the experiments; Contributed reagents, materials, analysis tools or data; Wrote the paper.

Ekoa Bessa Armel Zacharie: Performed the experiments; Analyzed and interpreted the data; Wrote the paper.

Messina Thérèse Raïssa: Performed the experiments; Analyzed and interpreted the data.

Onana Vincent Laurent: Conceived and designed the experiments; Performed the experiments; Analyzed and interpreted the data.

Ndjigui Paul-Désiré: Conceived and designed the experiments; Performed the experiments.

### Funding statement

This work was partly supported by the Department of Earth Sciences of the Faculty of Science and the Department of Biological Sciences of the Higher Teacher Training School (University of Yaoundé I, Cameroon).

### Data availability statement

Data included in article/supplementary material/referenced in article.

### Declaration of interests statement

The authors declare no conflict of interest.

### Additional information

No additional information is available for this paper.
